# Is Europe on the Way to Sustainable Development? Compatibility of Green Environment, Economic Growth, and Circular Economy Issues

**DOI:** 10.3390/ijerph20021078

**Published:** 2023-01-07

**Authors:** Simona Andreea Apostu, Iza Gigauri, Mirela Panait, Pedro A. Martín-Cervantes

**Affiliations:** 1 Department of Statistics and Econometrics, Faculty of Economic Cybernetics, Statistics and Informatics, Bucharest University of Economic Studies, 010552 Bucharest, Romania; 2 Institute of National Economy, 050711 Bucharest, Romania; 3 School of Business, Computing and Social Sciences, St. Andrew the First-Called Georgian University, 0179 Tbilisi, Georgia; 4 Cybernetics, Economic Informatics, Finance and Accounting Department, Faculty of Economic Studies, Petroleum-Gas University of Ploiești, 100680 Ploiești, Romania; 5 Department of Financial Economics and Accounting, Faculty of Economics and Business Sciences, University of Valladolid, 47002 Valladolid, Spain

**Keywords:** green environment, economic growth, circular economy, granger causality, Europe, sustainability

## Abstract

The challenges imposed by climate change and the limited nature of resources generate paradigm shifts at the level of economic, social, and environmental policies and strategies. Promoting the principles of sustainable development and the circular economy is a priority worldwide. Thus, the motivation of this research is to explore the European countries’ path toward sustainable development by analysing the relationship between green environment, economic growth, and circular economy issues. In order to explore this relationship in the case of European countries, the analysis takes into consideration specific variables: final energy consumption, GDP, capital gross fixed capital formation, greenhouse gas emissions, SOx emissions, NOx emissions, and generation of municipal waste per capita. This study is focused on the period 2009–2020 for 31 European countries, with data being provided by Eurostat and World Bank databases. The panel data analysis was used in order to examine the relationship between a green environment, economic growth and a circular economy. The results of the study suggest that gross fixed capital formation and total greenhouse gas emissions lead to decreasing generation of municipal waste; instead, final energy consumption, GDP, SOx emissions and NOx emissions generate an increase in the generation of municipal waste. The novelty of our paper consists of associating green environment, economic growth, and circular economy in the case of European countries, the results allowing the proposal of economic policy measures to favor the green transition process considering the potential of the circular economy.

## 1. Introduction

A circular economy, known as a cost-effective economy contributing to sustainable development, encompasses efficient use and recycling of resources. The concept, first introduced in 1990 and originated by Pearce and Turner [1], integrates Reduction, Reuse, and Recycle (3R) principles to reduce emissions and increase the shift towards green environment management. Accordingly, resources must be used to create value for people and the planet, and hence, products must be recyclable, energies must be generated from renewable sources, economic activities must protect ecology, and support the well-being of society [2,3]. In this context, economic growth and sustainable development become related to the circular economy, which surpasses transforming waste into resources by recycling but also offers a new mindset of achieving growth while protecting natural resources.

Circular Economy suggests extending the lifecycle of materials, diminishing pollution caused by the production process, and disconnecting economic growth from environmental damage [4,5,6]. The concept includes efficient use of energy and resources as well as avoiding waste as landfills occupy lands and threaten the environment requiring large financial investments [2]. To solve this issue, governments in European countries strive to explore various approaches to a circular economy to achieve sustainable development. 

Sustainable development considers economic, ecological, technological, and social pillars simultaneweously [7,8,9]. Economic growth exerts pressure on the environment causing environmental pollution and degradation. Emissions, waste, and energy consumption are critical concerns of sustainable development, which can be addressed by the circular economy. The circular economy is promoted as a catalyst of green growth, which connects economic growth with environmental protection. However, gross domestic product (GDP) is associated with the increase in environmental impact hindering green growth [10]. 

Sustainable development puts an emphasis on social well-being and environmental sustainability rather than a mere pursuit of GDP growth. Still, not a single country on a global scale satisfies the needs of its people in a sustainable way, including the sustainable use of resources [11]. The economy worldwide has been becoming less sustainable, although the scientific evidence insists on an urgent solution toward sustainable transformation [12]. The exponential growth must be balanced with sustainability as the planet has limits. On the one hand, the Sustainable Development Goals (SDGs) address protecting nature, conserving the ecosystem, and managing resources sustainably, and on the other hand, economic growth is required (SDG 8) to conquer poverty and eradicate hunger. Therefore, studies investigating the issue from different angles to find solutions are timely and particularly important. 

The motivation of this research is to explore the European countries’ path towards sustainable development by analysing the relation between green environment, economic growth, and circular economy issues. Therefore, the variables related to the circular economy and municipal waste are investigated through panel data analysis. The purpose of this research is to explore the correlation between a green environment, economic growth, and a circular economy. It aims to analyse the relationship between variables associated with the green environment, economic growth, and circular economy concepts. In particular, the paper strives to provide answers to questions of whether or not the final energy consumption, GDP, and various types of emissions lead to the waste increase. This research investigates the relationship between the green environment, economic growth, and circular economy in European countries in the time period between 2009–2020. The results display the complexity of the sustainable development process. The energy transition and the circular economy are two essential dimensions that are addressed by the countries of the European Union. Therefore, the contribution of various stakeholders can enable the objectives established by the Green Deal and the implementation of the new circular economy action plan. The results of the study allow the development of economic policies to facilitate the green transition process considering the potential that the circular economy has. Based on the results, the governments of EU countries can implement sustainable environmental and energy policies leading to the transition towards a circular economy. The study presents theoretical and practical implications for sustainable development and the transition to a circular economy. Based on the research results, future studies will explore the regional approach to the circular economy in the core countries and the new member countries of the European Union in terms of waste, energy consumption, and emissions.

## 2. Literature Review

### 2.1. Energy Consumption, Emissions, and Economic Growth

The use of energy has increased globally from 1995 to 2015 [13]. The energy sector is essential to create wealth and encouraging the economic development of a country [14,15]. The intensification of economic activity has generated an increase in the demand for energy, and energy producers are looking for new sources of energy to protect the environment, considering the environmental challenges that energy production and consumption entails. Humanity is going through a new energy transition that involves a decrease in the consumption of fossil fuels and the predominant use of renewable energy, a fact that generates numerous economic, social, and technical challenges but also business opportunities that are reaped even by the companies in the oil and gas field [16,17].

Energy is the main factor for sustainable development goals to achieve inclusive economic growth [18]. However, sustainable development can be ensured through sustainable and efficient use of energy while reducing greenhouse gas emissions [19,20,21,22]. SDG 3 and SDG 11 include targets regarding air pollution’s effects on people.

Consequently, the EU is striving to move towards green and clean energy, and the European Union Emissions Trading System takes constant efforts to reduce emissions in EU member countries. In addition, the setup of the Energy Union is an important step done by European countries in this race toward energy transition [17,23,24,25]. European citizens agree with the decision to reduce carbon emissions and favor renewable energy sources based on the efforts of public authorities to promote a just transition equitable for consumers, local communities, and companies [26,27,28,29,30,31,32,33]. While renewable energy consumption has a positive impact on reducing CO_2_ emissions, its significance is less visible due to economic growth and consumption of non-renewable energy sources [13].

Although the environmental Kuznets curve (EKC) proposed that “environmental degradation increases with per capita income during the early stages of economic growth, and then declines with per capita income after arriving at a threshold” [34], the subsequent studies have not confirmed this relationship [34]. Interestingly, research results propose that although economic growth comes at the expense of ecological degradation, especially, in the initial phase of development, in fact, carbon emissions can be reduced by income growth in many countries [35,36,37,38,39,40,41,42].

Empirical research using the multivariate model to analyse the correlation between economic growth and energy consumption in China from 1960 to 2007 confirmed that the level of carbon emissions and increased energy consumption do not induce economic growth [34]. This result paves the way for governmental interventions towards reducing carbon emissions and energy consumption without preventing economic development [34]. Such regulations can mitigate climate change and contribute to the sustainability agenda.

The research conducted in South Korea demonstrated that energy consumption decreased during the COVID-19 pandemic, but it has increased in residential facilities [43]. Earlier studies in the country revealed a bidirectional relationship between economic growth and energy consumption [44].

Previous multicounty studies indicated the interrelationship between economic growth, energy consumption, and carbon emissions. In particular, economic growth and energy consumption cause carbon emissions, whilst economic growth is more influences the level of emissions in developing countries, but this relationship is not confirmed in developed economies [45]. Energy consumption leads to economic growth in developing economies, while such a correlation is not established in developed countries [45]. 

In the case of Vietnam, Tang et al. [46] found that energy consumption, FDI and capital stock positively influence economic growth, from energy consumption to economic growth, running unidirectional causality. For OECD countries, renewable energy significantly and positively influences the economic [47]. In addition, in the case of 29 OECD countries for the period from 1990 to 2013, Gozgor et al. [48] considered the panel autoregressive distributed lag, and the panel quantile regression estimations highlighted that economic complexity, but also both the non-renewable and the renewable energy consumption are positively associated with economic growth. 

In fact, studies examining the correlation between economic growth and energy consumption are focused on different countries/regions with different backgrounds [49,50,51,52], and the results are controversial. Some of them confirmed the relationship between economic growth and energy consumption [35,53,54,55]. Other scholars have found no relationship between economic development and energy consumption [21,56,57], while some results illustrate a bidirectional relationship between the two variables [58,59,60,61]. With regard to multicounty studies, the results revealed the unidirectional relationship between economic growth and energy consumption [62,63,64,65]. However, other studies discovered no relationship when analysing the data from multiple countries [64]. Still, other scholars found a bidirectional correlation between energy consumption and economic growth [65,66,67,68,69].

Kasman and Duman [70] investigated the relationship between economic growth, energy consumption, and CO_2_ emissions in new EU members and candidate countries from 1992 to 2010. They found a short-term causality between energy consumption and carbon emissions, economic growth and energy consumption, and a bidirectional relationship between economic growth and energy consumption [70]. 

Previous research results demonstrated the relationships between energy consumption and carbon dioxide emissions. In particular, there is a unidirectional relationship between energy consumption and carbon emissions [53,55,65,71] and a bidirectional relationship between energy consumption and carbon dioxide emissions [59,63,66,72,73]. Thus, many studies confirmed that energy consumption leads to economic growth suggesting that the energy sector is a key driver of economic development as energy facilitates the achievement of economic goals.

### 2.2. Municipal Waste and Circular Economy

Municipal waste management (MWM) is of particular importance in the transition to a circular economy. On the one hand, MWM focuses on reducing the environmental impact of waste, and on the other hand, it pursues the recycling and reuse of certain materials. Owing to the fact that it reduces the pressure on natural resources in some situations, such as e-waste, specialists consider it to be an urban mine for certain industrial and precious metals [74,75,76]. In addition, MWM generates business opportunities, which implies the appearance of new companies in the business ecosystem of each country, as well as new jobs [77,78,79]. This is especially the case in the European Union due to the economic policy [80,81,82,83]. The European Commission published n 2020 the new circular economy action plan that presents a key part of the European Green Deal, and that emphasises the prominence of municipal waste management [84]. So, there are strong connections between waste management and the circular economy due to business opportunities used by different entrepreneurs in order to recycle and reuse certain materials [81,85,86,87].

The amount of waste generated is increasing in the European states as well as worldwide and consequently, the quantity of municipal waste per capita is also growing [83]. Annually, approximately 11.2 billion tonnes of solid waste is gathered globally, making up 5% of greenhouse gas emissions [88]. While municipal waste is about 10% of the total waste generated, reducing atmospheric emissions at landfills can prevent environmental pollution and improve the ecosystem [89]. 

Municipal waste mostly ends up at landfills in many European countries, which involves risks to the environment and people’s health [89]. According to the data from 2015, municipal solid waste in the European Union amounted to 477 kg per capita/year, of which 46% was recycled or composted [90]. However, the amount of waste has been increasing with the rise in living standards and consumption. In 2020, 4.8 tonnes of waste were generated per EU inhabitant, while 39.2% of waste was recycled and 31.3% landfilled [91].

The circular economy, against the background of sustainable development, strives to utilise natural resources in an efficient way by reducing waste and recycling in order to provide sufficient resources for future generations [19,85,92,93,94,95]. To stimulate the circular economy in the EU, not only municipal waste management needs to be implemented, but also ensuring sustainable energy consumption, as well as environmental protection, must be achieved [93,96].

Prior studies indicate that the implementation of the circular economy can promote economic growth and increase GDP, as well as provide environmental protection and reduce the deployment of natural resources [97]. The study results conducted by Sverko Grdic et al. [97] demonstrated the correlation between GDP and municipal waste. The EU countries with the highest GDP—Luxembourg and Denmark—are also the countries with the greatest amount of municipal waste generation per capita [97]. A large-scale multicounty study performed by Malinauskaite et al. [98] in the European states exposed that there is no relationship between GDP and waste generation, as the greatest amount of waste was generated in the UK and Italy with the higher GDP, and in Greece and Latvia with the lowest GDP per capita [98]. In addition, Norway, with the highest GDP rate, generates waste below the EU average [98]. It should be noted that GDP increase and population growth lead to the expansion of waste recycling [99]. The municipal waste sector represents a valuable input source for waste recyclable re-industrialisation from a circular economy perspective [100]. The main aim of European Union waste management directives implies the prevention of waste through reuse, recycling, other recovery, and disposal, i.e., circular economy [101].

In this context, we formulated the following hypothesis:

**Hypothesis** **H1.**  *The circular economy is significantly influenced by a green environment and economic growth*.

## 3. Data and Methodology

### 3.1. Methodology

In order to analyse the relationship between the green environment, economic growth and circular economy, we proposed to use the panel data analysis. Panel data consists of time-series observations across cross-sectional units [102]. Thus, it implies both a spatial and a temporal dimension, characterising the cross-sectional units over a given period of time [103].

For panel data, we used panel regression analysis. The first step in order to estimate the regression model is verifying the stationarity of the variables. The stationarity implies the existence of a unit root for which we have three models (without intercept or time trend, with intercept but no time trend, and including intercept and time trend) [104].

In order to test the stationarity were performed Levin, Lin and Chu—LLC [105], Im, Pesaran & Shin W-Stat—IPS [106], ADF-Fisher Chi-Square, and PP-Fisher Chi-Square tests. According to the panel unit root test, for all variables is accepted the alternative hypothesis. Thus, the variables are stationarity at a level.

The panel data model includes three different methods: Common constant, Fixed effects, and Random effects. To select between random and fixed effects estimation methods, the Hausmann test is used [107]. The null hypothesis implies no correlation between independent variables and error terms [108], and the alternative hypothesis assumes there is a statistically significant correlation between independent variables and error terms in panel data [109].

The econometric model for panel regression is described as follows:

y_i_ = β_0_ + β_1_ (x_1_)_it_ +β_2_ (x_2_)_it_ + β_3_ (x_3_)_it_ + β_4_ (x_4_)_it_ + β_5_ (x_5_)_it_ + β_6_ (x_6_)_it_ + ε_it_ (1)

where y represents the dependent variable and x_1_, x_2_, x_3_, x_4_, x_5_, and x_6_ are the independent variables. β_0_, β_1_, β_2_, β_3_, β_4_, β_5_, and β_6_ are the parametric coefficients, i − 1, …, 31 represents the number of countries and t − 1, …, 12 represents the time frame, ε being the error term.

Robustness checks imply heteroskedasticity of residues, the dependence of residues between the panels and dependence of residues between the panels and can be conducted by the Wooldridge autocorrelation test [110] and Wald test (heteroskedasticity of residues), Pesaran test (dependence of residues between the panels) and Greene heteroscedasticity test [111] and LM test (dependence of residues between the panels). The advantage of panel regression analysis is that it considers the information between each pair of time points, not only the beginning and the end of the sample [112].

### 3.2. Data

In order to explore the relationship between green environment, economic growth and circular economy in the case of European countries, we included the following variables in the analysis: final energy consumption, GDP, capital gross fixed capital formation, greenhouse gas emissions, SOx emissions, NOx emissions, and generation of municipal waste per capita. Generation of municipal waste per capita reflects a circular economy, GDP and capital gross fixed capital formation reflect economic growth, and final energy consumption, greenhouse gas emissions, SOx emissions, and NOx emissions reflect a green environment. Data are provided by Eurostat and World Bank databases, described in Table 1.

The study was realised using panel data of 372 observations over the period 2009–2020, the sample consisting of 31 European countries due to data availability. The European countries were selected because they have a certain level of economic development, and the authorities have intense concerns about promoting the principles of sustainable development [35,37,38,39,42,113]. Moreover, at the level of the European Union, public and private efforts regarding the energy transition and the circular economy are greater considering the legal regulations promoted in the member countries, regulations that can be a source of inspiration for other countries as well. For the empirical analysis, the program EViews 12.0 was used (IHS Markit: London, UK).

## 4. Empirical Results

To examine the sample characteristics descriptive analyses of the data were conducted. A summary of descriptive statistics of each variable for the entire sample can be seen in full in Table 2.

**Figure 1:**
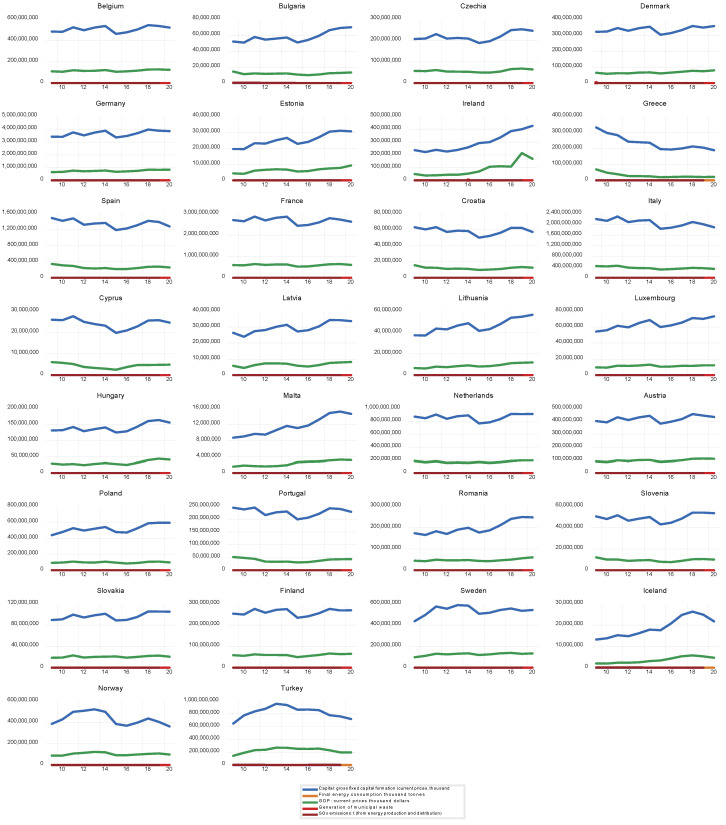
Variable Trends.

As can be observed in Figure 1, there are differences between countries regarding our variables. Although the EU countries are developing the concept of circular economy and are introducing that idea into practice toward SDGs, the level of circular economy development differs between individual countries.

To answer the research objectives regarding the relationship between circular economy, economic growth and green environment in European countries, we used the panel data equation model as follows:

GMW_it_ = *β*_it_ + *β*_1_ FEC_it_ + *β*_2_ GDP_it_ + *β*_3_ CGFCF_it_ + *β*_4_ SOx_it_ + *β*_5_ NOx_it_ + *β*_6_ GHG_it_ + ε_it_

The dependent variable is represented by the generation of municipal waste in order to reflect the circular economy. The explanatory variables included in the regression equations are Final Energy Consumption, GDP, Gross Fixed Capital Formation, SOx emissions, Nitrous oxide emissions and Total greenhouse gas emissions. To verify the correlation existing among the variables, we employed Pearson correlation (Table 3).

According to Table 3, the generation of municipal waste shows a positive correlation with gross fixed capital formation, final energy consumption, GDP, nitrous oxide emissions and total greenhouse gas emissions, indicating that all variables except SOx emissions can influence a generation of municipal waste in case of the countries in the sample.

In order to test the stationarity, an LLC test was used [105], IPS test [106], Fisher ADF test, and Fisher PP test [114] are suitable due to the assumption of individual unit root process in each cross-section series. The results of the unit root test are listed in Table 4, according to which we claim that the variables are stationary.

To estimate the influence of the variables: Final Energy Consumption, GDP, Gross Fixed Capital Formation, SOx emissions, Nitrous oxide emissions and Total greenhouse gas emissions on the generation of municipal waste, from a cross-sectional and longitudinal perspective, we considered two models: with fixed effects and random effects. According to the Hausman specification test (Table 5), there is a significant difference regarding the statistical results of random and fixed effects. Therefore the fixed effects model was selected for estimating the model. 

The fixed effects model obtained explaining 99.8% of the generation of municipal waste is defined by gross fixed capital formation, final energy consumption, GDP, nitrous oxide emissions, total greenhouse gas emissions, and SOx emissions (Table 6).

In order to test homogeneity, the results (Table 7) support as pertinent the estimation of the generation of municipal waste based on gross fixed capital formation, final energy consumption, GDP, nitrous oxide emissions, total greenhouse gas emissions, and SOx emissions in case of using models with fixed effects. The variables’ influence on the generation of municipal waste over time is constant. As Prob is below 0.05, the null hypothesis of homogeneity is accepted. Thus, the generation of a municipal waste evaluation model is unique and representative of European countries.

The results obtained from the GWM_it_ estimation using the fixed effects model are presented in Table 8. The results indicated that the Final Energy Consumption, GDP, Gross Fixed Capital Formation, SOx emissions, Nitrous oxide emissions and Total greenhouse gas emissions have a significant influence on the generation of municipal waste, considering a probability of 90%.

Therefore, the regression equation can be written as follows:

GMH_it_ = 4483.68 + a_i_ + d_t_ − 4.83 ∗ 10^−6^ GFCF_it_ + 0.151 FEC_it_ + 1.80 ∗ 10^−5^ GDP_it_ − 0.012 GHG_it_ + 128.66 NOx_it_ + 0.001 SOx_it_. (2)

where a_i_ are the fixed effects determined by the individual size of the countries (differences between countries in terms of generation municipal waste), and dt represents the fixed effects determined by the temporal dimension (differences between years regarding generation municipal waste in the European countries).

Thus, using the fixed effects, it is assumed that the influence of the variables on the generation of municipal waste is similar for all countries, regardless of the period (2009–2020). The robustness checks highlighted no autocorrelation and heteroscedasticity problems.

According to Equation (2), gross fixed capital formation and total greenhouse gas emissions lead to decreasing generation of municipal waste; instead, final energy consumption, GDP, NOx emissions and NOx emissions generate an increase in the generation of municipal waste. Thus, the generation of municipal waste is significantly influenced by variables representing renewable energy and economic growth, validating Hypothesis 1.

## 5. Discussion and Conclusions

The research presented in the paper analysed the relationship between green environment, economic growth, and circular economy in the European countries in the time period between 2009–2020 through the following variables: Final energy consumption, greenhouse gas emissions, SOx emissions, NOx emissions reflecting green environment; GDP, capital gross fixed capital formation reflecting economic growth; and Generation of municipal waste per capita reflecting circular economy. As the research results demonstrated, gross fixed capital formation and total greenhouse gas emissions can lead to decreasing generation of municipal waste, while final energy consumption, GDP, SOx emissions and NOx emissions cause an increase in the generation of municipal waste. Our results are relatively similar to what we found in the literature. Jafari et al. [115] indicated in Bahrain a unilateral causality running from urban population, economic growth, capital and energy consumption to the environment and from economic growth to energy consumption, emissions and capital. According to Soytas and Sari [21], in the case of Turkey’s carbon emissions, Granger causes energy consumption, but the vice versa is not confirmed. Instead, between income and emissions, there is no long-run causality. Fei et al. [116] studied the relationship between GDP and energy consumption in China using data from 1985 to 2007, showing a positive long-run cointegration. Using data from a sample of 20 net energy importers and exporters from 1971 to 2002, Mahadevan and Asafu-Adjaye [117] highlighted a bidirectional causality between economic growth and energy consumption in the developed countries, both in the short and long run, while in the developing countries energy consumption stimulates growth only in the short run. Using data from 35 OECD countries over the period 2000–2014, Ozcan et al. [118] found that economic growth and energy consumption patterns contribute to the countries’ environments. In order to investigate the nexus between energy consumption, economic growth, and CO_2_ emission in Pakistan, Khan et al. [119] used annual data from 1965 to 2015 using ARDL. The results indicated that energy consumption and economic growth increase the CO_2_ emissions in Pakistan both in the short run and long run. In the case of G7 countries, Cai et al. [63] found that clean energy consumption causes real GDP per capita for Canada, Germany, and the US, and CO_2_ emissions cause clean energy consumption for Germany.

The results of the study demonstrate the complexity of the sustainable development process, the energy transition and the circular economy being two dimensions that are intensively addressed by the countries of the European Union. The involvement of stakeholders including public authorities, companies and consumers ensures the achievement of the bold objectives established by the Green Deal also by the new circular economy action plan.

### 5.1. Theoretical and Practical Implications

The results of the study allow the development of economic policies to facilitate the green transition process considering the potential that the circular economy has. Based on the results, the governments of EU countries should implement sustainable environmental and energy policies leading to the transition towards a circular economy. In this regard, non-renewable energy sources need to be replaced by renewable energy sources. Likewise, green procurement policies can be implemented, which encourage industries to focus on the sustainable production process and give incentives to decrease emissions and reduce energy consumption [120,121]. Furthermore, municipal waste should be recycled rather than end up in landfills. Since countries with lower GDP have more tendency to send the waste to landfills as this process is cheaper than recycling, the governments can introduce financial aid, additional regulations, or landfilling tax to inspire the reuse of waste, for example, for producing energy [102].

In addition, citizens should be motivated to adopt sustainable practices by reducing waste and energy consumption in accordance with the modern paradigm of reuse, recycling, and recovery [122,123]. Businesses and society can initiate sorting and recycling waste, as well as saving energy and reducing harmful emissions [101,124]. By adopting circular economy principles, companies can utilise waste as a resource [101]. However, it is noteworthy that the EU countries are different in terms of industries or economic development, resulting in various approaches towards the circular economy.

Moreover, innovative technologies can be utilised in municipal waste sorting with automation and robots [125]. In this sense, artificial intelligence can enable efficient and sustainable management of waste to achieve zero waste objectives. Besides, new technologies support environmentally friendly results by monitoring and reducing pollutant emissions [47,126].

Collaboration between various stakeholders is essential to achieve sustainable development [127], and the setup of public-private partnerships is a proper solution for such a complex issue [128,129]. Sustainability education is one of the key enablers in the transition to a circular economy [93,130,131,132,133]. Through education, sustainable entrepreneurship can be stimulated, new technologies can be discovered, and innovative business models and strategies compatible with the circular economy principles can be developed.

### 5.2. Conclusions

The level of economic development generates an intensification of concerns regarding the protection of the environment but also the increase of social inclusion, with significant results being recorded on the European continent. The countries of the European Union stand out as international promoters of sustainable development, this process being a politically driven one, which is why the results recorded are very good. The existence of common regulations and the integration of economic policies at the regional level facilitate the transition to the green economy through two main pillars, namely the energy transition and the circular economy. GHG emissions, waste, and energy consumption are critical issues of sustainable development, which can be addressed by the circular economy. Recycling and reuse of certain materials generate a reduction in the consumption of natural resources, lowers energy consumption, and waste management reduces the impact of human activity on the environment. In addition, waste management provides new business opportunities that ensure the direct involvement of citizens and companies in supporting sustainable development. The solutions offered by the circular economy are intensively followed by the European authorities but also by companies, especially in the context of the events in Ukraine that have generated an awareness of the need to decrease the dependence on imports of raw materials and the vulnerability of the European Union to certain countries such as Russia.

Although the literature maintains research results investigating the relationship between economic growth, energy consumption, and emissions, Waheed et al. [45] still indicate the need to explore this relationship in terms of different country groups. Thus, this research explored the correlation between the green environment, economic growth, and circular economy in European member states from 2009 to 2020 by analysing the related variables.

### 5.3. Limitations and Future Research

This scientific study has certain limits generated by choice of the analysis period and the countries selected by the authors. The authors chose the countries of the European Union reflecting the considerable progress made by this entity in the process of promoting sustainable development by adopting specific legal regulations through which bold targets and specific instruments were established regarding the energy transition, digital transition and circular economy. However, at the level of the European Union, there are major differences between the member countries regarding the progress made, which is proven by statistical data. For this reason, future research will focus on the regional approach to the energy transition—the circular economy relationship between the core countries and the new member countries of the European Union. Moreover, among the new member countries, the former communist countries of Central and Eastern Europe could be the subject of a separate study considering certain similarities regarding the level of development and also certain cultural peculiarities generated by the communist regime. The authors also consider the use of other indicators to reveal the need for e-waste recycling, taking into account both the dramatic impact on the environment through the faulty management of this waste and also the saving of mineral resources that can be achieved by recycling electrical and electronic equipment.

## Figures and Tables

**Table 1:** Dataset definition.

Variables	Definition	Unit	Source
FEC	Final Energy Consumption	Thousand tons of oil equivalent	https://ec.europa.eu/eurostat/databrowser/view/TEN00124__custom_2684073/default/table?lang=en (accessed on 15 November 2022)
GDP	Current GDP	US$	https://data.worldbank.org/indicator/NY.GDP.MKTP.CD (accessed on 15 November 2022)
GFCF	Gross Fixed Capital Formation	%GDP	https://data.worldbank.org/indicator/NE.GDI.FTOT.ZS (accessed on 15 November 2022)
SOx	SOx emissions	Sulphur oxides	https://ec.europa.eu/eurostat/databrowser/view/ENV_AIR_EMIS__custom_2721609/default/table?lang=en (accessed on 15 November 2022)
NOx	Nitrous oxide emissions	thousand metric tons of CO_2_ equivalent	https://data.worldbank.org/indicator/EN.ATM.NOXE.KT.CE (accessed on 15 November 2022)
GHG	Total greenhouse gas emissions	thousand metric tons of CO_2_ equivalent	https://data.worldbank.org/indicator/EN.ATM.GHGT.KT.CE (accessed on 15 November 2022)
GMW	Generation of municipal waste	Thousand tonnes	https://ec.europa.eu/eurostat/databrowser/view/env_wasmun/default/table?lang=en (accessed on 15 November 2022)

**Table 2:** Main characteristics of the series.

Variables	Mean	Std. Dev.	Min	Max
FEC	34,618.80	46,515.20	362.50	209,923.1
GDP	541,716,375.63	839,831,465.28	8,696,367.00	3,980,000,000.00
GFCF	114,410,709.01	173,863,104.17	1,590,068.00	838,000,000.00
SOx	171,962.76	434,837.90	160.00	2,668,470.00
NOx	3.38	4.85	0.02	18.74
GHG	109,430.55	159,313.50	1397.47	784,475.60
GMW	8544.94	12,427.49	132.00	52,133.00

Source: Authors’ computation using Eviews.

**Table 3:** Pearson’s correlation among variables.

	GMW	GFCF	FEC	GDP	GHG	NOx	SOx
GMW	1	0.941	0.977	0.944	0.951	0.963	0.485
GFCF	0.941	1	0.974	0.992	0.914	0.895	0.192
FEC	0.977	0.974	1	0.971	0.967	0.951	0.354
GDP	0.944	0.992	0.971	1	0.902	0.888	0.242
GHG	0.951	0.914	0.966	0.902	1	0.943	0.426
NOx	0.963	0.895	0.951	0.888	0.943	1	0.497
SOx	0.485	0.192	0.354	0.242	0.426	0.497	1

Source: Authors’ computation using Eviews.

**Table 4:** Unit root tests for the full sample.

Variables	Levin, Lin & Chu		Im, Pesaran & Shin W-Stat		ADF-Fisher Chi-Square		PP-Fisher Chi-Square	
	Statistic	Prob.	Statistic	Prob.	Statistic	Prob.	Statistic	Prob.
FEC	−3.213	0.001	−1.589	0.056	71.637	0.145	95.196	0.003
GDP	−3.339	0.000	0.100	−1.281	80.919	0.037	97.654	0.002
GFCF	−3.094	0.001	−0.489	0.312	59.439	0.496	71.465	0.148
SOx	−1.951	0.026	1.747	0.960	45.872	0.911	148.526	0.000
NOx	−5.047	0.000	−1.604	0.054	82.245	0.030	103.408	0.000
GHG	−9.555	0.000	−2.886	0.002	105.485	0.000	55.285	0.648
GMW	−1.561	0.059	0.764	0.778	68.868	0.204	91.096	0.006

Source: Authors’ computation using Eviews.

**Table 5:** Correlated random effects-Hausman test.

Test Summary	Chi-Sq. Statistics	Chi-Sq. d.f.		Prob.
Cross section random	140.938	6		0.000
Cross-section random effects test comparisons				
Variables	Fixed	Random	Var (Diff.)	Prob.
GFCF	−4.83 ×10^−6^	9.53 × 10^−7^	−5.071	0.000
FEC	0.151	0.017	8.939	0.000
GDP	1.80 × 10^−5^	2.85 × 10^−6^	6.314	0.000
GHG	−0.012	0.004	−3.213	0.002
NOx	128.657	82.505	1.559	0.120
SOx	0.001	0.000	2.631	0.009

Source: Authors’ computation using Eviews.

**Table 6:** Statistics on the fixed-effects model evaluation.

Regression Model Statistics	
Sum of Squares of Errors	75,880,804
Standard error of regression	511.525
Coefficient of Determination (R^2^)	0.998

Source: Authors’ computation using Eviews.

**Table 7:** Testing the homogeneity hypothesis based on the F test.

F Test for Fixed Effects		
Number of Fixed Effects	F Value	Prob (F-Statistic)
30	5272.565	0.0000

Source: Authors’ computation using Eviews.

**Table 8:** Estimation of the regression model parameters.

Variables	Coefficients	Std. Error	t-Statistic	Prob.
GFCF	−4.83 × 10^−6^	9.53 × 10^−7^	−5.071	0.0000
FEC	0.151	0.017	8.939	0.0000
GDP	1.80 × 10^−5^	2.85 × 10^−6^	6.314	0.0000
GHG	−0.012	0.004	−3.213	0.0000
NOx	128.657	82.505	1.559	0.120
SOx	0.001	0.000	2.631	0.009
Intercept	4483.683	518.662	8.645	0.0000

Source: Authors’ computation using Eviews.

## Data Availability

Not applicable.
